# The Principles of Surgery

**Published:** 1845-04

**Authors:** 


Art. IV.—*The Principles of Surgery.* By James Miller, F.R.S.E., F.R.C.S.E., Professor of Surgery in the University of Edinburgh, Surgeon to the Royal Infirmary, &c. Philadelphia: Lea & Blanchard. 1845. pp. 525. 8vo.

Most of our readers are aware that the author of this book fills the place vacated by the death of one of the most celebrated men of this century; and they share doubtless in the curiosity that we felt to know what manner of man the authorities of the University of Edinburgh had selected to succeed Bell, from among the number of distinguished individuals with which that city abounds. His name when first announced was certainly not generally familiar to the profession abroad, whatever it may have been at home; and we cannot help thinking that Prof. Miller was led to put forth his volume of "Principles" to show that the mantle of Bell had fallen on one not unworthy of it, nor yet unworthy to be the compeer of Syme, Lizars, and a host of others.

Be this, however, as it may, we confess ourselves pleased with the book. It is one of the few whose title expresses neither more nor less than what it contains; it is one essentially of *principles*, and these of such a sort as make us think the author is a skilful and discriminating surgeon.

The first section is devoted to *Elementary Disease* under the heads of *perverted action of the bloodvessels, of the nerves, of the absorbents; suppuration; ulceration; mortification.* In the second section, the author treats of elementary disease occurring in the different tissues, which gives us *perverted vascular action in the integuments, in the bones and joints, in the arteries, veins, lymphatics* and *nerves.* Section third is occupied with *perverted nutrition,* as seen in the different tumors; section fourth discusses *injuries,* including *wounds, burns* and *scalds, effects of cold, fracture, dislocation, &c.*

The longest and most elaborate chapter in the book is that wherein the author discusses the subject of inflammation. In this he shows that he has been not an unmindful observer of what is going on around him, but has profited by the progress of physiology and pathology, as elucidated in the past few years by chemistry and the microscope. Although his account of the successive changes which take place in inflammation are, we believe, correct, he assumes a somewhat novel ground as to what shall be understood by the term. It has, in the opinion of the author, been made to include "too wide a range," involving actions the most dissimilar and opposite. Hence he limits the term "to what is essentially morbid," and lays down, as the characteristic of true inflammation, the formation of pus.

Granting that "practical confusion and tendency to error" have resulted from the views more commonly inculcated, it seems to us that instead of diminishing, he has greatly added to them; that in trying to avoid Scylla, he has gone recklessly right over into Charybdis. According to the author's view, a man's brain may be overlaid with lymph, and a section of it disclose numerous rosaceous, or otherwise colored spots; but it is not inflammation, it is only "active congestion." The pleura and lung may be firmly united by false membrane; but it is only active congestion. The trachea may be lined by fibrinous exudation; yet it is only active congestion. The substance of the lung may be rendered impervious to air—may be hepatized; once more it is only active congestion. The peritoneum and the serous covering of the intestines may be covered by lymph, as in a case which we recently saw; but still it is only active congestion. If this be not 'tendency to error,' and 'practical confusion' worse confounded, we would very much like to know what is. At this rate there would scarcely be any such thing as death from inflammation—we wish it were so. But in truth, the author is not consistent with himself. He proposes, as we just stated, to limit the term inflammation to that which is "essentially morbid." All very well; we do not object to this. We take the phrase, too, in his own sense, and in his own language, as meaning that which is "at variance with healthy function and structure." Now how many instances are there in which the effusion of fibrin is compatible with healthy function and structure? Nay, we will go further; in how many instances is the effusion of *serum* not at variance with healthy function and structure?

The fifth chapter, which includes ulcers, while it gives additional evidence of his scientific skill in managing disease, is liable to similar criticism. The necessity of a right classification and the positive harm arising from a wrong one, are properly urged, and then he proceeds to divide them into "1. The Simple Purulent, or Healthy Sore. 2. The Weak. 3. The Scrofulous. 4. The Indolent. 5. The Irritable. 6. The Inflamed. 7. The Sloughing. 8. The Phagedænic. 9. The Sloughing Phagedæna." Why this is as bad as Astley Cooper's twelve sorts of ulcer, and only not quite so bad as Everard Home's six principal varieties with as many sub-varieties, besides varicose ulcers, ulcers from irritation of the nails, and those attending various malignant diseases! If we speak of scrofulous ulcer, why not of scorbutic ulcer, arthritic ulcer, or an ulcer with any sort of prefix derived from actual or supposed peculiarity of constitution? Every body knows, thanks to Abernethy, that ulcers vary according to the varying states of the general system, and that they require both general and local treatment; but if all the differences thus derived are to be described, how many kinds of ulcer would we have? would there be any end to description?

But we came to praise the author not to find fault with him; and the arrangements of our printer have left us little room for either. The defects such as we have noticed, do not detract greatly from the absolute merits of the book, upon which we are disposed to set high value. The chapters on aneurism, hæmorrhage, wounds, and on the management of the inflammatory process, we think will more than warrant us in our opinion.

We make an extract from the latter chapter, on the use of mercury, simply because it happens to suit our limits. It will serve as a specimen of the author's compact and vigorous style, at the same time that it conveys valuable practical information.

"*Mercury.*—The mercurial is often the preferable form of purge at the outset of treatment—calomel, followed by jalap, for example; causing copious exhalation from the intestinal mucous membrane, promoting a free flow of bile, and—if that secretion be part of the fuel by whose intra-combustion animal heat is maintained, as chemistry has of late hinted—obviously tending to lower the febrile increase of temperature. But it is not as a purgative that mercury is chiefly antiphlogistic; not when it passes quickly through, but when it is retained in the primæ viæ, absorbed thence into the system, and lays hold of this, exerting on it a specific effect; the systemic seizure being usually indicated by fœtor of the breath, tenderness of the gums, and rawness of the mouth, which, if the introduction of the mineral be continued, advances to complete salivation. But as it was not the purgation, so is it not the salivation which we usually desire. Mercury, gradually introduced into the system, seems to exert a tonic effect on both the extreme blood-vessels and the lymphatics, that is on the exhalants and the absorbents; thus preventing or limiting impending effusion, and at the same time expediting the removal of that which has been already exuded. The affection of the gums is not of itself valuable, but only as showing that the impregnation of the system by the mineral is so far advanced, as to be equal to the effecting of this result—truly antiphlogistic. Besides, mercury is supposed to act directly on the blood, affecting the red corpuscles, as well as assisting in the removal of the anormal proportion of its fibrin.

"From its power of at once limiting and removing effusion it is very plain how valuable must be its administration in all inflammatory affections of important internal organs, whose functions must seriously suffer by any considerable change of structure, however temporary; or when texture is extremely delicate—even slight effusion producing much disorder, and hard to be recovered from—as in the iris, and synovial membrane. When such parts are becoming truly inflamed we give mercury with much eagerness, desirous that its constitutional effect should be both speedy and complete. But he is a sadly thoughtless and reprehensible practitioner who throws in mercury with a loose hand and a careless eye for inflammations in general—real or supposed—regardless of the risk thereby encountered of hopeless ruin to the system at no very distant date, by the remote consequences of wanton and uncalled-for mercurialism. It is fortunate for us that such risk need not be dreaded by the wary surgeon, who not only gives no mercurial course unless such be demanded, but also inquires diligently into the circumstances connected with that demand, ere he admits it to be just and true. When satisfied on this point, he hesitates no longer; but proceeds to his duty of saving vitally important texture and function at all hazards, comforted by a belief, well-grounded on experience, that by such inflammations there is engendered a tolerance of mercury, both present and future.

"The best form of exhibition is calomel, usually combined with opium, in the form of pill; two grains of the former, with half a grain of the latter, repeated every hour or every two hours, according to the haste with which we desire to affect the system. The opium prevents the mercury being wasted as a purge, and insures its internal reception by absorption, while itself has a beneficially sedative result on both the nervous and vascular systems. It has lately been proposed—in accordance with a laudable desire to obtain the constitutional effect at the least possible cost of mercury—to give calomel in very minute doses, often repeated; as the twelfth of a grain every hour; absorption being supposed to take place very readily and fully from minute doses—as is exemplified in the internal use of arsenic. Such caution is much to be commended, and such doses are highly allowable in cases of no great urgency, either as to intensity of action or importance of texture involved; but the old experienced dose, as above stated, is far more trust-worthy in the true inflammatory emergency. Should calomel and opium be found to disagree, a convenient substitute may be found in the hydrargyrum cum cretâ with Dover's powder. When it is desirable to affect the system with extreme rapidity, or when the ordinary mode of exhibition is peculiarly tardy, the desired result may be accelerated by rubbing in a mercurial ointment or liniment on the inside of the thighs, in the axillæ, or over the part affected.

"Mercury, however, let it ever be remembered, is only subsidiary and second to bleeding as an antiphlogistic. The intensity of the vascular action must be first broken by loss of blood; the remainder is then well dealt with by the mercury. But should the latter go single handed to the contest, it is sure to fail; harm is done instead of good; the inflammation and its accompanying fever are both augmented, as can be readily understood, when it is borne in mind that the direct effect of the medicine is stimulant or tonic to the capillaries.

"The time, then, at which we are to commence the exhibition of mercury for antiphlogistic purposes is after blood-letting; we desist when the gums have been 'touched,' as the ordinary phrase is, showing the attainment to systemic seizure; or we may often cease from its use at a still earlier period, the symptoms which demanded it having satisfactorily given way. Should the disease, on the contrary, prove obstinate, even after ptyalism has been induced, the mercury may be cautiously resumed, so as to maintain this until recedence or change in the symptoms occur; but in no case of mere inflammation is full, far less sustained, salivation at all necessary.

"In all cases, before enjoining its administration, it is well to inquire as to the existence or not of idiosyncrasy regarding it; whether the patient is easily affected, or otherwise; whether liable to the troublesome eczema, or to the dangerous erethismus.

"Should mercury both gripe and threaten to purge, notwithstanding combination with opium or hyoscyamus, it is well that the doses be given in some bulky vehicle. In non-inflammatory cases—as certain forms of the venereal disease—such disagreeable tendencies are readily avoided by giving the mercury immediately after the ordinary meals.

"Locally, mercury is of use, in the form of plaster or ointment, applied to the part affected; but the proper time for its employment is still later than that of the internal exhibition. It is meet to oppose, not the action itself, but rather its results on texture. All acuteness of action must have been previously subdued by the earlier and more appropriate remedies; and then mercurial inunction, by its tonic and stimulant effect on the blood-vessels and absorbents, may happily restore the tone of the former, as yet dilated and weak, and prone to continuance of effusion; while it rouses the absorbents to an increased duty, that effusion may be removed and normal condition of texture restored. But at an earlier period, the same application, now so beneficial, could not fail, by stimulating the blood-vessels, to aggravate the action and the changes of structure to which it tends."

We will only repeat, by way of conclusion, that the student will find in the book a body of sound principles, forcibly and concisely expressed. With this volume and some such work as Fergusson's *Practical Surgery,* his rough way may be made more smooth.

As to the style in which the book is published, it is sufficient to say it is from the press of Lea & Blanchard.

